# Case Report: The first Mainland Chinese case of *CTLA4* c.529dupT haploinsufficiency with a 38-year diagnostic delay

**DOI:** 10.3389/fimmu.2026.1899668

**Published:** 2026-09-16

**Authors:** Xiang Gu, Jian Guo, Lei Li, Yi Xia, Xiaohui Lv, Bing Li

**Affiliations:** 1 Department of Respiratory and Critical Care Medicine, Shanghai Fourth People’s Hospital, Tongji University School of Medicine, Shanghai, China; 2 Department of Radiology, Second Affiliated Hospital of Naval Medical University, Shanghai, China

**Keywords:** CTLA4 haploinsufficiency, hypogammaglobulinemia, phenotypic heterogeneity, sirolimus, whole−exome sequencing

## Abstract

Cytotoxic T-lymphocyte-associated protein 4 (CTLA4) haploinsufficiency with autoimmune infiltration (CHAI) is an autosomal dominant condition featuring both immunodeficiency and autoimmunity. We describe the first published mainland Chinese patient harboring the heterozygous CTLA4 c.529dupT frameshift variant. While multiple CHAI cases have been documented in mainland China, this specific variant has not been reported in this population to date. The patient developed recurrent thrombocytopenia, recurrent respiratory infections and hypogammaglobulinemia at age 2. She was diagnosed with immune thrombocytopenia (ITP) at age 15. The disease subsequently progressed to Evans syndrome, alongside complications including chronic diarrhea, cerebral vasculitis, biliary tract infection and interstitial pneumonia. She received standard anti-infective therapy, combined glucocorticoids and immunosuppressants, as well as intravenous immunoglobulin (IVIG) replacement; however, these interventions yielded only limited and temporary benefits. After 38 years of disease progression, a primary immunodeficiency was suspected. Whole-exome sequencing (WES) verified the diagnosis of CHAI. Treatment with sirolimus led to amelioration of infection and diarrheal symptoms. This case exhibited phenotypic differences from prior patients carrying the identical variant. Our observations expand the known phenotypic spectrum of this variant and offer clinical references for the management and genetic testing of patients presenting with early-onset multisystem autoimmunity and immunodeficiency.

## 1 Introduction

Cytotoxic T-lymphocyte-associated protein 4 (CTLA4) is a co-inhibitory molecule expressed on regulatory T cells and activated T lymphocytes. It maintains immune homeostasis by restraining T-cell activity (1–4). Heterozygous loss-of-function (LoF) *CTLA4* variants give rise to CTLA4 haploinsufficiency with autoimmune infiltration (CHAI), with penetrance reaching 60%–70% (3). Clinically, CHAI presents with combined immunodeficiency and excessive immune activation (5–7). CHAI was initially recognized and formally named in 2014 (1). To date, more than 220 cases have been documented internationally based on a 2021 systematic review (8), with most reported in European and American cohorts. Data from Chinese populations remain scarce, with only small case series and isolated reports published to date (9–15). Here we report a mainland Chinese patient carrying the *CTLA4* c.529dupT frameshift variant, which has not been previously reported in mainland China. The patient had a 38-year diagnostic delay from symptom onset to genetic confirmation. We describe her clinical trajectory, laboratory and imaging features, genetic findings and treatment response, and compare her phenotype with published cases to delineate the heterogeneity of this variant.

## 2 Case presentation

### 2.1 Clinical timeline

The patient was a 40-year-old female admitted with recurrent fever, severe pneumonia and chronic diarrhea. Her disease spanned 38 years, with key clinical events summarized in **Table 1**.

**Table 1:** Summary of key clinical events.

Age at event	Major manifestations/diagnosis	Management/outcome
2 years	Recurrent respiratory infections, thrombocytopenia	Corticosteroids + IVIG; symptoms relieved
15–19 years	Refractory ITP, herpes zoster, splenomegaly	Corticosteroids + IVIG + prednisone + vincristine/cyclosporine
20–23 years	Evans syndrome, pulmonary and cutaneous mucormycosis, subarachnoid hemorrhage, subdural hematoma	Corticosteroids + IVIG + amphotericin B/itraconazole/voriconazole + blood transfusion
27–28 years	Cerebral vasculitis, left limb weakness (grade 2), Evans syndrome, respiratory and cutaneous infections	Corticosteroids + IVIG + cyclophosphamide
32–34 years	Common bile duct stones, acute cholangitis, obstructive suppurative cholangitis, post-cholecystectomy, Evans syndrome	ERCP + stone extraction + ENBD + stent placement + cholecystectomy + corticosteroids + IVIG
38 years	Interstitial lung disease with infection, renal insufficiency, Evans syndrome, post-cholecystectomy	Anti-infective therapy + nebulization
40 years	Severe pneumonia, Evans syndrome, cerebral vasculitis, chronic diarrhea, renal insufficiency, hyperuricemia, post-cholecystectomy	Anti-infective therapy + IVIG + albumin; CHAI confirmed by WES; sirolimus 1 mg daily + IVIG with improved diarrhea
41 years	CHAI, post-cholecystectomy, history of cerebral hemorrhage, history of gastrointestinal bleeding	Sirolimus 2 mg daily + IVIG; diarrhea resolved

IVIG, intravenous immunoglobulin; ITP, immune thrombocytopenia; ERCP, endoscopic retrograde cholangiopancreatography; ENBD, endoscopic nasobiliary drainage; CHAI, CTLA4 haploinsufficiency with autoimmune infiltration; WES, whole-exome sequencing.

Symptoms first appeared at age 2, with recurrent lower respiratory tract infections and persistent thrombocytopenia. At age 15, she was diagnosed with immune thrombocytopenia (ITP), accompanied by herpes zoster, chronic watery diarrhea and splenomegaly. At age 20, superimposed autoimmune hemolytic anemia led to a revised diagnosis of Evans syndrome; her platelet nadir reached 0 × 10^9^/L, complicated by gastrointestinal hemorrhage, subarachnoid hemorrhage and left foot gangrene.

Invasive cutaneous and pulmonary aspergillosis and mucormycosis developed sequentially at age 23. At age 27, left hemiparesis occurred, and cerebral vasculitis was confirmed by neuroimaging. Recurrent cholecystitis and obstructive suppurative cholangitis manifested at age 32, followed by new-onset interstitial lung disease at age 38.

On admission at 40 years of age, she presented with fever, tachycardia and hypotension in the context of severe pneumonia and renal insufficiency. Multidisciplinary case review followed by whole-exome sequencing ultimately confirmed CHAI disease. Over the preceding decades, she had received multiple lines of immunosuppressive and supportive therapies, including systemic glucocorticoids, cyclophosphamide, cyclosporine and repeated IVIG infusions, which yielded only transient symptom control and frequent disease flares. Both parents were healthy, with no family history of primary immunodeficiency or autoimmune disorders.

### 2.2 Physical examination on admission

Physical examination on admission identified scattered pigmented lesions over both lower extremities and a low body mass index consistent with chronic disease-related wasting. Auscultation of the lungs revealed diminished bilateral breath sounds with fine inspiratory crackles. Cardiac, abdominal and neurological examinations were otherwise unremarkable.

### 2.3 Laboratory findings

#### 2.3.1 Hematological parameters and inflammatory markers (**Table 2**)

Admission blood tests showed neutrophil-predominant leukocytosis, moderate anemia and persistent thrombocytopenia. Inflammatory markers including C-reactive protein (CRP), serum amyloid A (SAA), procalcitonin (PCT) and interleukin-6 (IL-6) were elevated at baseline, declined with antimicrobial treatment, and rebounded after influenza A infection (**Table 2**).

**Table 2:** Changes in hematological parameters and inflammatory markers.

Date	WBC (×10^9^/L)	Neutrophil (%)	Hb (g/L)	PLT (×10^9^/L)	CRP (mg/L)	SAA (mg/L)	PCT (ng/mL)	IL–6 (pg/mL)
2025-01-14	12.05	74.0	90	60	15.99	305.24	26.71	43.58
2025-01-17	2.56	52.8	76	65	21.08	9.25	6.49	28.12
2025-01-20	2.77	60.6	71	82	6.82	16.26	0.88	73.39
2025-01-23	1.35	69.1	73	55	18.08	16.05	0.38	49.21

WBC, white blood cell; Hb, hemoglobin; PLT, platelet; CRP, C-reactive protein; SAA, serum amyloid A; PCT, procalcitonin; IL-6, interleukin-6.

#### 2.3.2 Immunological workup

Immune workup before IVIG replacement revealed profound hypogammaglobulinemia: IgG 0.08 g/L, IgA <0.23 g/L, IgM <0.21 g/L. Complement component 3 (C3) was decreased, while C4 remained normal. Flow cytometry showed markedly reduced natural killer (NK) cells and B lymphocytes, with CD4^+^ and CD8^+^ T cell counts within normal ranges. After IVIG infusion, serum IgG and C3 normalized, but IgA and IgM remained low. No serum autoantibodies were detected (**Table 3**).

**Table 3:** Immunoglobulin, complement and lymphocyte subset levels.

Parameter	Value	Unit	Reference range	Date	Timing	Remarks
IgG	0.08	g/L	7.0-16.0	2025-01-15	Pre-IVIG	Low
	12.1	g/L	7.0-16.0	2025-01-30	Post-IVIG	Normal
IgA	<0.23	g/L	0.7-4.0	2025-01-15	Pre-IVIG	Low
	<0.23	g/L	0.7-4.0	2025-01-30	Post-IVIG	Low
IgM	<0.21	g/L	0.4-2.3	2025-01-15	Pre-IVIG	Low
	<0.21	g/L	0.4-2.3	2025-01-30	Post-IVIG	Low
C3	0.63	g/L	0.9-1.8	2025-01-15	Pre-IVIG	Decreased
	0.98	g/L	0.9-1.8	2025-01-30	Post-IVIG	Normal
C4	0.19	g/L	0.1-0.4	2025-01-15	Pre-IVIG	Normal
	0.32	g/L	0.1-0.4	2025-01-30	Post-IVIG	Normal
CD4^+^ T cells	559.52	/μL	400-1600	2025-01-15	Pre-IVIG	Normal
CD8^+^ T cells	285.05	/μL	200-800	2025-01-15	Pre-IVIG	Normal
NK cells	33.82	/μL	136.29-880.40	2025-01-15	Pre-IVIG	Decreased
B lymphocytes	21.44	/μL	91.53-498.00	2025-01-15	Pre-IVIG	Decreased

IgG, immunoglobulin G; IgA, immunoglobulin A; IgM, immunoglobulin M; C3/4, complement component 3/4, NK cells, natural killer cells.

#### 2.3.3 Microbiological testing

Serum galactomannan and 1,3-β-D-glucan assays were negative. Sputum and bronchoalveolar lavage fluid cultures and nucleic acid tests yielded no pathogenic pathogens. Influenza A virus infection was confirmed by nucleic acid testing 6 days after admission at 40 years of age.

### 2.4 Radiological findings

Representative brain MRI and chest CT images are shown in **Figure 1**. Brain MRI obtained at 27 years of age identified multifocal subcortical lesions in the right frontoparietal and bilateral parieto-occipital regions, which resolved after pulse methylprednisolone alongside improved left hemiparesis.

**Figure 1:**
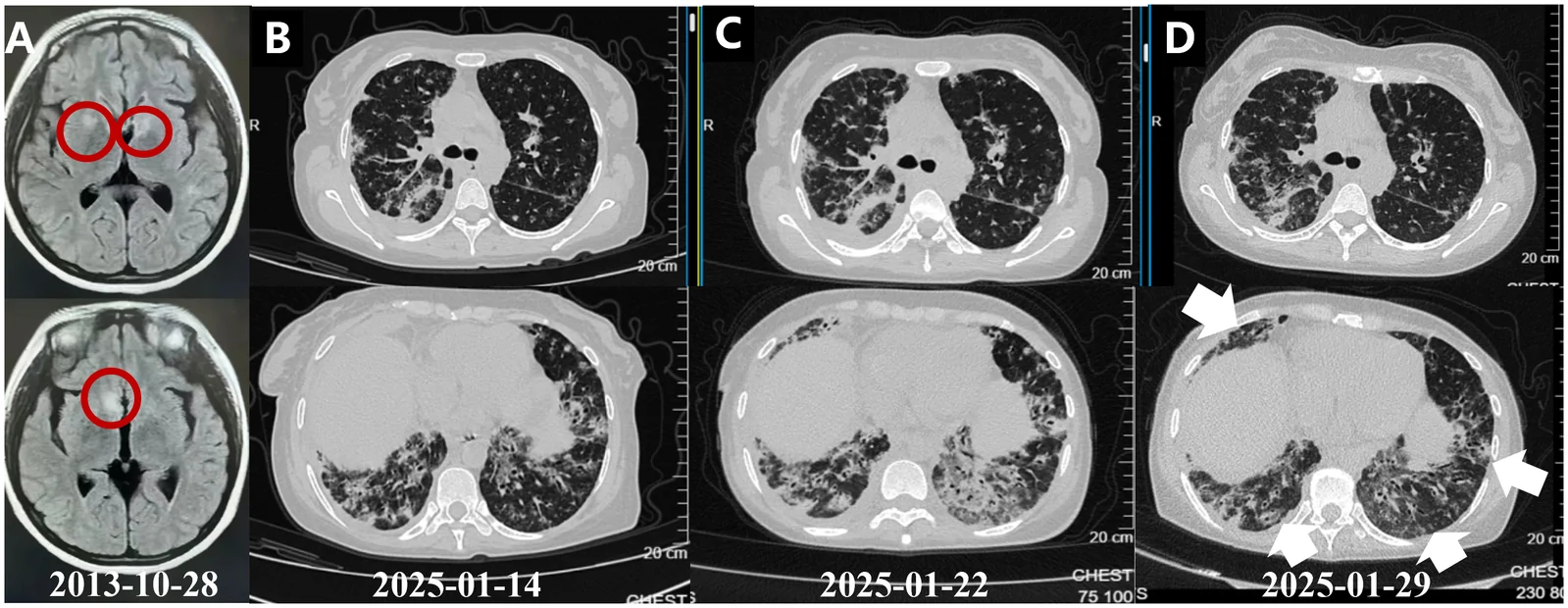
Representative imaging findings of the patient. **(A)** Brain magnetic resonance imaging (MRI) at 27 years of age: Subcortical lesions (red circles) in the right frontoparietal and bilateral parieto-occipital regions, hypointense on T1 and hyperintense on T2/FLAIR. **(B)** The first chest computed tomography (CT) after admission at 40 years of age: Bilateral peribronchial acinar nodules with partial consolidation. **(C)** Chest CT at 40 years of age, 8 days after anti-infective treatment initiation: Unresolved pulmonary infiltrates. **(D)** Chest CT at 40 years of age, following 5-day IVIG at 10 g daily: Significant lesion absorption (white arrows: resolving inflammation). MRI, magnetic resonance imaging; CT, computed tomography; FLAIR, fluid-attenuated inversion recovery; IVIG, intravenous immunoglobulin.

Chest CT obtained at 40 years of age revealed bilateral acinar nodules along bronchovascular bundles with partial consolidation and air bronchograms. Pulmonary infiltrates responded poorly to antimicrobials but improved markedly after IVIG. Abdominal ultrasound confirmed splenomegaly.

### 2.5 Initial hospital management

The patient received empirical anti-infective therapy with levofloxacin, piperacillin–tazobactam and meropenem, plus baloxavir marboxil for influenza A. Pneumonia did not resolve significantly. After 5 days of IVIG (10 g daily), pulmonary lesions gradually absorbed and chronic diarrhea improved notably.

## 3 Materials and methods

### 3.1 Whole-exome sequencing and bioinformatic analysis

Given the patient’s atypical multisystem phenotype, whole-exome sequencing was preferred over a targeted gene panel, enabling comprehensive coverage of both established and putative PID-associated genes. Genomic DNA was extracted from peripheral blood leukocytes of the proband and both parents. Sequencing libraries were constructed with the VAHTS Universal DNA Library Prep Kit for Illumina V4 following the manufacturer’s protocol. WES was performed on the DNBSEQ-T7 platform with a mean coverage depth of 37.12×. The full workflow is summarized in **Supplementary Figure 1**.

Raw sequencing reads were aligned to the human reference genome GRCh38/hg38 via the Aliyun dna-variant-calling pipeline v1.1. Single-nucleotide variants and small insertions/deletions were called using GATK v4 and annotated with VEP v113 against the ncbiRefSeqCurated transcript set and ClinVar database. Copy number variations were detected with CNVpytor v1.3. Candidate variants were filtered by a gnomAD East Asian allele frequency < 0.01 and prioritized for autosomal dominant inheritance patterns consistent with the clinical phenotype. A heterozygous *CTLA4* variant was validated by Sanger sequencing in the proband and both parents, and its pathogenicity was classified according to ACMG/AMP 2015 guidelines.

### 3.2 Genetic findings

Whole-exome sequencing identified a heterozygous frameshift variant in *CTLA4* (NM_005214.5: c.529dupT, p.Tyr177LeufsTer2). Sanger sequencing confirmed the variant in the proband and its absence in both parents, verifying a *de novo* germline origin (**Figure 2**). The variant was classified as pathogenic per ACMG criteria (PVS1, PM2, PM6, PP3).

**Figure 2:**
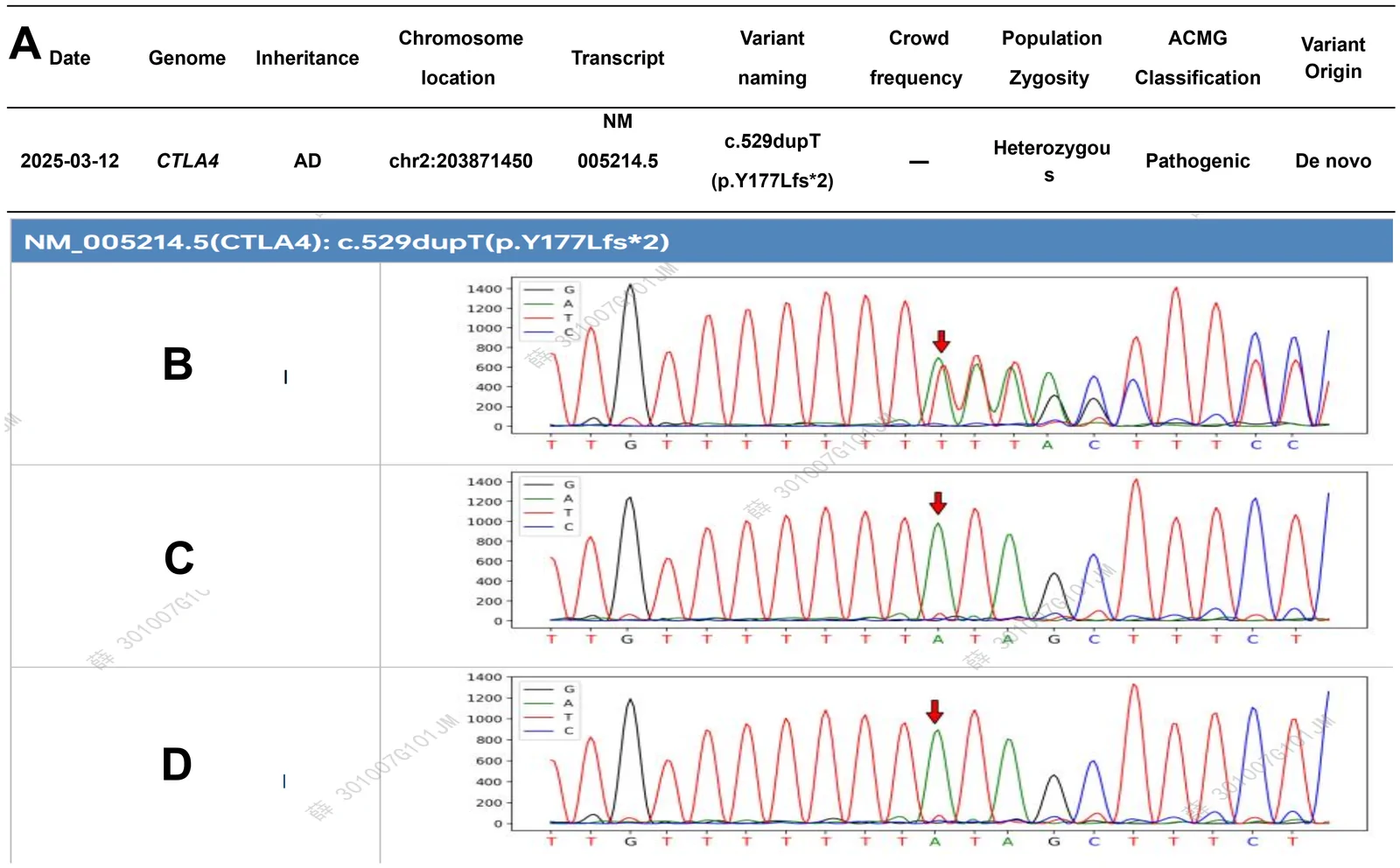
Whole-exome sequencing and Sanger sequencing validation of the *CTLA4* variant. **(A)** WES identified a heterozygous frameshift variant in CTLA4 (NM_005214.5: c.529dupT, p.Tyr177LeufsTer2) in the proband. **(B–D)** Sanger sequencing chromatograms: **(B)** Proband: Heterozygous c.529dupT variant; **(C)** Father: Wild-type; **(D)** Mother: Wild-type. CTLA4, cytotoxic T-lymphocyte-associated protein 4; WES, whole-exome sequencing.

Family pedigree analysis is shown in **Figure 3**. The patient is an only child, married with no offspring. No other family members exhibited immune dysregulation, and both parents carried wild-type *CTLA4* alleles. Given the single affected individual with unaffected parents, familial penetrance cannot be assessed in this two-generation pedigree.

**Figure 3:**
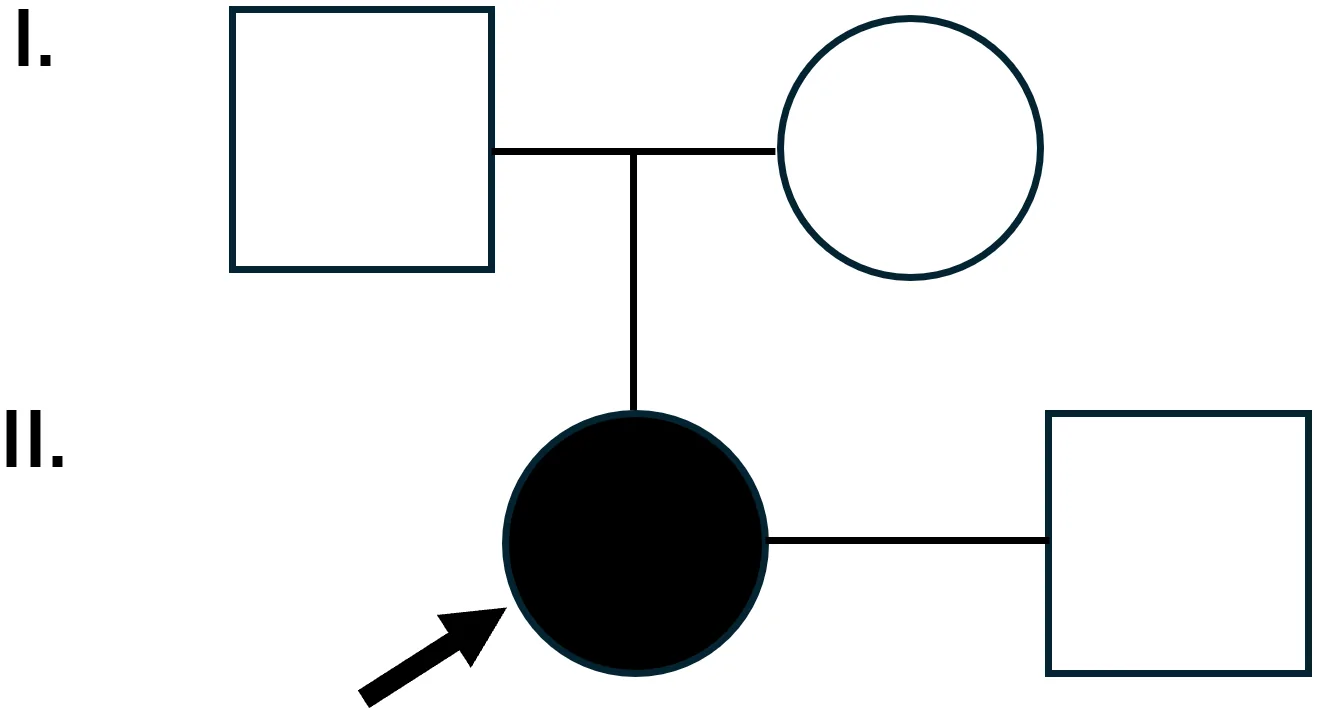
Two-generation family pedigree of the proband.

Squares indicate male individuals; circles indicate female individuals. The filled circle marked with an arrow represents the affected proband. Both parents carry wild-type *CTLA4* alleles, confirming *de novo* origin of the c.529dupT variant. No other family members showed signs of immune dysregulation.

## 4 Literature search strategy

To place this molecular diagnosis and systematically define the phenotypic spectrum of CTLA4 haploinsufficiency associated with this variant, we performed a systematic literature search of PubMed, CNKI, Wanfang and VIP databases for reports published up to May 2026. Search terms included “CTLA4”, “c.529dupT”, “haploinsufficiency”, “CHAI”, “China”, “Chinese” and “mainland”. Original case reports and cohort studies with confirmed sequencing data were included; review articles, unvalidated conference abstracts and cases from Taiwan, Hong Kong and Macao were excluded. A systematic review identified all previously reported mainland Chinese CHAI cases (summarized in **Supplementary Table 1**), none of which carried the *CTLA4* c.529dupT variant.

## 5 Treatment and follow-up

At 40 years of age, oral sirolimus was initiated at 1 mg daily for worsening diarrhea, with dose titration to 2 mg daily guided by therapeutic drug monitoring (target trough 10–15 ng/mL (16, 17)). After 3 days of treatment, diarrhea frequency decreased from >10 to 4–5 episodes daily; complete resolution of diarrhea was achieved after approximately one month, along with a marked reduction in infectious episodes. Mild hypertriglyceridemia occurred as an adverse event and was managed with dietary modification alone.

At follow-up at 41 years of age, the patient remained stable on sirolimus 2 mg daily plus monthly IVIG replacement.

Prior to sirolimus initiation, the patient had only received routine mandatory childhood vaccines, with no prior influenza or pneumococcal vaccination. At 40 years of age, while maintained on sirolimus and IVIG, she received one dose of inactivated influenza vaccine, after which self-reported respiratory infection episodes decreased. However, vaccine-specific serological testing was not performed, so quantitative humoral response data are unavailable and a causal relationship cannot be confirmed.

## 6 Discussion

This report describes the first published mainland Chinese patient carrying the *CTLA4* c.529dupT pathogenic variant, with an exceptionally long 38-year diagnostic delay. The case recapitulates core CHAI features (1–4)—early-onset autoimmune cytopenias, hypogammaglobulinemia, recurrent infections and lymphoproliferation—while exhibiting rare severe complications that expand the known phenotypic spectrum of this variant.

Only one prior case carrying the identical *CTLA4* c.529dupT variant has been reported, in a Middle Eastern patient (18). Key differences between the two cases are outlined in **Table 4**. The previously reported patient had adult-onset disease, an inflammatory/allergic immune profile, and predominant renal and dermatological autoimmunity. In contrast, our patient had early childhood onset, profound humoral immunodeficiency, and severe neurological and biliary complications. These differences highlight the marked variable expressivity of this frameshift variant, likely modified by ethnic genetic background and environmental factors.

**Table 4:** Comparative clinical and genetic characteristics of patients carrying the *CTLA4* c.529dupT variant.

Characteristic	Present case	Reported case (Deeb et al. (18))
Ethnic background	East Asian (mainland China)	Middle Eastern
Variant origin	*De novo* (both parents wild-type)	Inherited heterozygous variant
Age at onset & diagnostic delay	Onset at 2 years; 38-year diagnostic delay	Onset at 21 years; diagnosis established within months
Baseline immune profile	Profound humoral immunodeficiency (low IgG/IgA/IgM; reduced B and NK cells)	Inflammatory/allergic phenotype (elevated IgE, eosinophilia)
Hematological manifestations	Autoimmune cytopenia (Evans syndrome, recurrent bleeding)	Chronic myeloid leukemia; mild-to-moderate thrombocytopenia
Major organ involvement	Cerebral vasculitis, relapsing cholangitis, interstitial lung disease	Anti-PLA2R-positive membranous nephropathy, bullous pemphigoid, Hashimoto thyroiditis
Severe infection pathogens	Opportunistic fungi (*Mucor*, *Aspergillus*)	Resistant bacteria (MRSA, *Pseudomonas aeruginosa*)
Targeted therapy	Sirolimus + monthly IVIG (abatacept unavailable locally)	Nilotinib, rituximab, cyclosporine + regular IVIG

Both cases share core features including splenomegaly, lymphadenopathy, chronic diarrhea, recurrent infections, and long-term dependence on corticosteroids and IVIG, suggesting consistent effects of the variant on lymphoid tissue and mucosal barriers, with final phenotypes modified by host immune background and comorbidities.

IVIG, intravenous immunoglobulin; MRSA, methicillin-resistant *Staphylococcus aureus*; NK, natural killer.

Consistent with large international cohorts (5, 8), our patient exhibited classic CHAI hallmarks including hypogammaglobulinemia, autoimmune cytopenias and chronic diarrhea. However, severe symptomatic cerebral vasculitis and recurrent obstructive suppurative cholangitis are rarely reported in published CHAI series, and have not been described in the recent mainland Chinese prospective cohort of 7 patients (9).

Notably, this 38-year diagnostic odyssey far exceeds the median 8-year delay documented in the recent mainland Chinese cohort (9). This gap stems from both historical and practical factors. The patient’s symptoms first appeared at 2 years of age, decades before CTLA4 haploinsufficiency was recognized as a distinct monogenic disorder and formally named CHAI in 2014 (1); no standardized diagnostic framework existed for this condition for most of her disease course. In addition, routine clinical practice lacked standardized genetic testing pathways for primary immunodeficiency in earlier decades, and WES was not widely accessible locally, which further prolonged the time to molecular diagnosis.

The combined infection-autoimmunity phenotype required differentiation from six primary immune disorders:

Immune dysregulation, polyendocrinopathy, enteropathy, X-linked (IPEX) syndrome: An X-linked disorder typically presenting with infantile enteropathy and endocrinopathies. Our patient is female with no history of childhood diabetes or eczema, making this diagnosis incompatible (19).
Lipopolysaccharide-responsive and beige-like anchor protein (LRBA) deficiency: An autosomal recessive condition with earlier onset and more severe malabsorption. The confirmed *de novo CTLA4* variant and autosomal dominant inheritance pattern exclude recessive LRBA deficiency (20).
Activated phosphoinositide 3-kinase delta syndrome (APDS): Characterized by prominent EBV-related lymphoproliferation and sinopulmonary infections, neither of which was observed in this patient (21).
STAT3 gain-of-function (STAT3 GOF) disease: Typically presents with short stature, early-onset diabetes and polyarthritis, all absent in our case (22).
Autoimmune lymphoproliferative syndrome (ALPS): Defined by persistent lymphadenopathy and expanded double-negative T cells. Routine immunophenotyping showed no such expansion, and lymphadenopathy was not a dominant feature (23).
Common variable immunodeficiency (CVID): Causes isolated hypogammaglobulinemia with milder autoimmune features, and cannot explain the severe cerebral vasculitis and multi-organ autoimmunity seen here (24).

Abatacept is recommended as first-line targeted therapy for CHAI (16), but was not accessible at our institution. In this case, sirolimus effectively controlled chronic diarrhea and reduced infection frequency, supporting its utility as an alternative targeted agent in settings without abatacept access. IVIG replacement remained essential for infection prevention (16).

This study has several limitations. No residual blood samples were available for ex vivo CTLA4 functional assays or high-resolution lymphocyte subset analysis. Follow-up after sirolimus initiation is approximately 7 months, so long-term efficacy and safety remain undetermined. Vaccine-specific antibody titers were not measured, precluding formal assessment of vaccine response.

## 7 Conclusion

We present the first confirmed mainland Chinese case of CHAI caused by the *CTLA4* c.529dupT *de novo* variant, with a 38-year disease course characterized by early onset, severe hypogammaglobulinemia, Evans syndrome, cerebral vasculitis and recurrent biliary infections. This case broadens the recognized phenotypic spectrum of the c.529dupT variant. Early WES testing for CHAI should be considered in pediatric patients with multisystem autoimmunity, recurrent infections and persistent hypogammaglobulinemia, even in the absence of a positive family history. Sirolimus is an effective, well-tolerated targeted option for CHAI patients in regions where abatacept is unavailable.

## Data Availability

The original contributions presented in the study are included in the article/**Supplementary Material**. Further inquiries can be directed to the corresponding author.

## Supplementary material

Supplementary Figure 1 WES experimental and bioinformatic workflow.
