# Supplementary material for: Case Report: The first Mainland Chinese case of CTLA4 c.529dupT haploinsufficiency with a 38-year diagnostic delay
Source: Front Immunol. 2026 Sep 16;17:1899668. doi: 10.3389/fimmu.2026.1899668 (PMC13623563; doi:10.3389/fimmu.2026.1899668)
Supplement: Supplementary file 1 [file Table1.docx]

Supplementary Material

# 1 Supplementary Table

**Supplementary Table S1. All published Chinese CHAI cases.**

| Patient ID | Sex | Age (years) | Key clinical manifestations | *CTLA4* variant | Region (mainland China) | Ref. |
| --- | --- | --- | --- | --- | --- | --- |
| 1 | Female | 24 | Anemia, cough, hypogammaglobulinemia, immune hemolysis, pulmonaryground-glass opacities, autoimmune hemolytic anemia, thrombocytopenia | c.155G>T(p.Gly52Val), heterozygous | Beijing | [9,15] |
| 2 | Male | 17 | Elevated CSF protein, epilepsy, limb tremors, paresthesia, seizures, speech difficulties | c.208C>T(p.Arg70Trp), heterozygous | Beijing | [10] |
| 3 | Male | 2 | Diarrhea, elevated DNT cells, fever, hepatosplenomegaly, hypogammaglobulinemia, immune hemolysis, ITP, lymphadenopathy, petechiae, pulmonary infection, purpura, recurrent infections, thrombocytopenia | c.151C>T(p.Arg51X), heterozygous | Beijing | [11] |
| 4 | Female | 20 | Arthritis, diarrhea, eczema, Evans syndrome, hepatosplenomegaly, arthralgia, photosensitive dermatitis | c.347T>A(p.Ile116Asn), heterozygous | Chongqing | [9] |
| 5 | Female | 60 | Fever, large granular lymphocyte leukemia, oral ulcers | c.347T>A(p.Ile116Asn), heterozygous | Beijing | [9] |
| 6 | Female | 19 | Anemia, diarrhea, eczema, lymphadenopathy, PNH-like manifestations, alopecia, positive intrinsic factor antibody | c.151C>T(p.Arg51Ter), heterozygous | Beijing | [9] |
| 7 | Male | 18 | Arthritis, cough, fever, hypogammaglobulinemia, arthralgia, lymphadenopathy, pulmonary infiltration | c.436G>A(p.Gly146Arg), heterozygous | Beijing | [9] |
| 8 | Male | 50 | Cough, diarrhea, erythema, hypogammaglobulinemia, rash, alopecia | c.151C>T(p.Arg51Ter), heterozygous | Beijing | [9] |
| 9 | Male | 23 | Diarrhea, erythema, pulmonary infection, rash, bronchial asthma, weight loss | c.208C>T(p.Arg70Trp), heterozygous | Shandong | [12] |
| 10 | Female | 6 | AIHA, ITP, recurrent infections, hypertensive encephalopathy | c.362_391del(p.Ala121_Lys130del), heterozygous | Nanchang, Jiangxi | [13] |
| 11 | Male | 22 | Positive AMA-M2, colonic ulceration, diarrhea, erythema, hypogammaglobulinemia, rash | c.538C>T(p.Leu180Phe), heterozygous | Qinghai | [9, 14] |
| 12 | Male | 13 | Diarrhea, lymphadenopathy, recurrent infections, hypogammaglobulinemia | c.211_212insTGACAGTGCTTCGGGCAGGC(p.Gln80Aspfs*4), heterozygous | Chongqing | [17] |

**Table note:** All variants listed are heterozygous germline variants. This table compiles all molecularly confirmed CHAI cases reported in mainland China to date. Patients 1,4–8, 11constitute the 7-patient prospective cohort described by Liu et al. ^[9]^; Patients 1 and 11 were additionally described in separate case reports ^[14, 15]^ and are not counted twice in the overall case tally. The c.529dupT variant detected in our patient is not present in any of these published cases, supporting that this is the first report of this specific variant in a mainland Chinese population.
